# Book Notices

**Published:** 1879-10-20

**Authors:** 


### *BOOK NOTICES.*

TRANSACTIONS OF THE COLLEGE PHYSICIANS OF PHILADELPHIA. Third series, vol. IV. Philadelphia: Lindsay & Blakiston.

This volume of the Transactions is one of much interest, containing a number of valuable papers. It contains 248 pages, large octavo, and is gotton out in the neat and elegant style for which the house of Lindsay & Blakiston is noted.

THE MULTUM IN PARVO REFERENCE AND DOSE BOOK, by C. HENRI LEONARD. Third edition, revised and enlarged. Detroit, southeast corner Gratio and Woodward Avenues. 1879.

This new edition of the Dose Book, says the author, contains the doses of over 2,500 preparations. My aim is that it shall be the most complete in this respect of any book issued. It is the out-of-the-way remedies, those little used, that the practitioner is apt to need a memory-poster on, and hence I have aimed to give as large a number as possible of this class of preparations in the Dose list. The doses of 225 new preparations have been added to the list in this edition.

POCKET THERAPEUTIC AND DOSE BOOK, with classification and explanation of medicines. Min. ane max. Doses in troy weights with their equivalents in the metric weights. Index and definition of diseases, with appropriate remedies. Genitive endings of all medicines and preparations given in Italics. Index of common and pharmaceutical names. Classification of symptoms. Poisons and their antidotes. Useful hints to the prescriber. By MORSE STEWART, JR., B. A., M. D. Second edition, revised and enlarged—handsomely bound in cloth, $2; handsomely bound in morocco, $1 30. Detroit, Mich.: Geo. D. Stewart. 1878.

This is a valuable work, indeed. Duodecimo, 264 pages.

